# Comprehensive Analysis Reveals Deoxyribonuclease 1 as a Potential Prognostic and Diagnostic Biomarker in Human Cancers

**DOI:** 10.7759/cureus.56171

**Published:** 2024-03-14

**Authors:** Loai F Eletr, Sahar H Ibnouf, Tanzeel A Salih, Hadba I Ibrahim, Mustafa I Mustafa, Nasma A Alhashmi, Mohamed Alfaki

**Affiliations:** 1 Computing and Bioinformatics, Faculty of Science, Port Said University, Port Said, EGY; 2 Pharmacy, Omdurman Islamic University, Khartoum, SDN; 3 Biotechnology, University of Bahri, Khartoum, SDN; 4 Zoology, Faculty of Science, University of Khartoum, Khartoum, SDN; 5 Internal Medicine, Sudan Medical Specialization Board, Khartoum, SDN; 6 Clinical Immunology, Sudan Medical Specialization Board, Khartoum, SDN; 7 Neurology, King Abdulaziz Medical City Jeddah, Jeddah, SAU; 8 Medical Parasitology, University of Gezira, Wad Madani, SDN; 9 Research, Sidra Medicine, Doha, QAT

**Keywords:** prognostic biomarker, diagnostic biomarker, kidney papillary renal cell carcinoma, kidney renal cell carcinoma, dnase1

## Abstract

Background: Deoxyribonuclease 1 (DNASE1) is an important gene associated with several cancers, including liver, bladder, and gastric cancer. It has been linked to autoimmune illnesses, including systemic lupus erythematosus, which may lead to cancer formation. However, the role of DNASE1 in cancer has not been studied.

Materials and methods: We performed a pan-cancer analysis using bioinformatics tools, including Tumor Immune Estimation Resource (TIMER), Gene Expression Profiling Interactive Analysis (GEPIA), and University of Alabama at Birmingham Cancer Data Analysis Portal (UALCAN) databases, Kaplan-Meier plotter, and cBioPortal, to investigate the expression of DNASE1 across various cancers as well as its association with immune infiltration and genetic alterations. Public datasets were used to validate DNASE1 expression in kidney renal clear cell carcinoma (KIRC) and kidney papillary renal cell carcinoma (KIRP) samples.

Results: DNASE1 was found to be highly expressed in many cancers, such as bladder urothelial carcinoma (BLCA), breast invasive carcinoma (BRCA), head and neck squamous cell carcinoma (HNSC), and was lowly expressed in other cancers, including KIRC, KIRP, and thyroid carcinoma (THCA). Additionally, TIMER results showed an association of DNASE1 with immune cell infiltration in KIRC and KIRP. Survival analysis indicated that high DNASE1 expression was associated with poor prognosis in KIRC. We also discovered that altered DNASE1 expression was related to poor prognosis in The Cancer Genome Atlas (TCGA) tumors.

Conclusion: DNASE1 could potentially be used as a prognostic and diagnostic biomarker for KIRC and as a diagnostic biomarker for KIRP.

## Introduction

Deoxyribonuclease 1 (DNASE1) has been linked to several types of cancer, including gastric, liver, and bladder cancers [1-3]. DNASE1 plays a role in apoptosis, which is critical in carcinogenesis. Furthermore, transcriptome analysis has highlighted the potential of genetic variations in DNASE1 as diagnostic and prognostic biomarkers for cancer [1]. DNASE1 mRNA expression is higher in tumoral tissues of bladder cancer than in the surrounding normal tissues [3]. Moreover, targeted expression of recombinant DNASE1 in ovarian cancer cells leads to their complete eradication without affecting healthy cells [2,4]. These findings indicate that DNASE1 may be useful for targeted cancer therapy. Furthermore, the relevance of DNASE1 in systemic lupus erythematosus (SLE) has been thoroughly investigated, revealing its involvement in autoimmune diseases [5,6]. DNASE1 is postulated to be responsible for removing DNA from nuclear antigens in regions with high cell turnover, thus preventing the initiation of SLE [6]. Moreover, DNASE1 loss in mice increases anti-DNA autoimmunity, highlighting its protective role [7]. DNASE1 has also been linked to other immunological illnesses, including Crohn's disease, and proposed as a possible biomarker for cancer detection [8,9]. DNASE1 expression was found to be higher in tumor cells of hepatocellular carcinoma than in normal liver tissues, indicating its potential role in liver cancer [10]. DNASE1 has been linked to several diseases, including cancer and autoimmune disorders. Its involvement in apoptosis, and its potential as a therapeutic target in cancer treatment, have sparked widespread attention in biomedical research. Despite this, the use of DNASE1 as a diagnostic or prognostic biomarker in cancer remains unique, as does its relationship with other immune cells such as macrophages, CD8+ T cells, dendritic cells, and neutrophils.

## Materials and methods

TIMER database

TIMER, also known as Tumor Immune Estimation Resource (https://cistrome.shinyapps.io/timer/), is a platform that assists computational biologists in analyzing the relationship between gene expression and the abundance of immune infiltrates in various cancers [11]. We utilized this method to determine the significance of DNASE1 expression in KIRC (kidney renal clear cell carcinoma) and KIRP (kidney renal papillary cell carcinoma), and to identify its correlation with the abundance of different immune cells. Additionally, we applied the Cox Proportional-Hazards Model to KIRC and KIRP where the expression of DNASE1 was significant.

GEPIA database

Gene Expression Profiling Interactive Analysis (GEPIA) is a web-based tool used for exploring gene expression patterns across different cancers using TCGA datasets (The Cancer Genome Atlas Program) (http://gepia.cancer-pku.cn/) [12]. The expression of DNASE1 was evaluated across several cancers using this tool to validate the expression pattern of DNASE1 from the TIMER database and examine its prognostic significance using Kaplan-Meier plots.

UALCAN database

UALCAN (University of Alabama at Birmingham Cancer Data Analysis Portal) is a user-friendly and powerful OMICS tool for cancer transcriptome analysis, including RNA-seq expression data from the TCGA dataset [13]. We established the expression of DNASE1 among various cancers to validate its expression pattern, which was obtained from both the GEPIA and TIMER databases. A Kaplan-Meier plot was generated to determine the correlation between DNASE1 expression and survival outcomes to assess its prognostic significance. Additionally, we identified the relationship between DNASE1 expression and different clinicopathological parameters, including age, sex, weight, cancer stage, and race, in KIRC and KIRP.

Kaplan-Meier plotter

Kaplan-Meier plotter (https://kmplot.com/analysis/) is an online survival analysis tool used to assess the relationship between the expression of every gene, including mRNA, miRNA, protein, and DNA, and the survival outcomes of patients in 3500+ samples from 21 tumor types. We investigated the correlation between the prognosis of patients with KIRC and KIRP and DNASE1 expression using Kaplan-Meier plots.

Genetic alterations analysis using cBioPortal

The cBioPortal for Cancer Genomics (https://www.cbioportal.org/) is an open platform for exploring and discovering genetic alterations using cancer genomic datasets [14]. It was employed to uncover the genetic alterations of DNASE1 in 10,967 tumor samples from 32 studies, specifically TCGA Pan Cancer Atlas Studies. 

Validation of DNASE1 expression

To validate our results, we obtained public datasets from the National Center for Biotechnology Information (NCBI) (https://www.ncbi.nlm.nih.gov/). We performed differential expression analysis using the GEO2R tool (https://www.ncbi.nlm.nih.gov/geo/geo2r/), an interactive web tool that allows researchers to compare two or more groups of samples to identify differentially expressed genes. This enabled us to identify the significance of DNASE1 in KIRC and KIRP. Differential expression profiles were visualized using volcano plots from the bioinformatics.com.cn platform (https://bioinformatics.com.cn/), which is an online platform mainly used for data analysis and visualization. Statistical analyses were performed based on the criteria of |Log2FC| > 1 and adj p-value < 0.05 for the identification of the differentially expressed genes.

## Results

DNASE1 expression across various types of cancer

**Figure 1:**
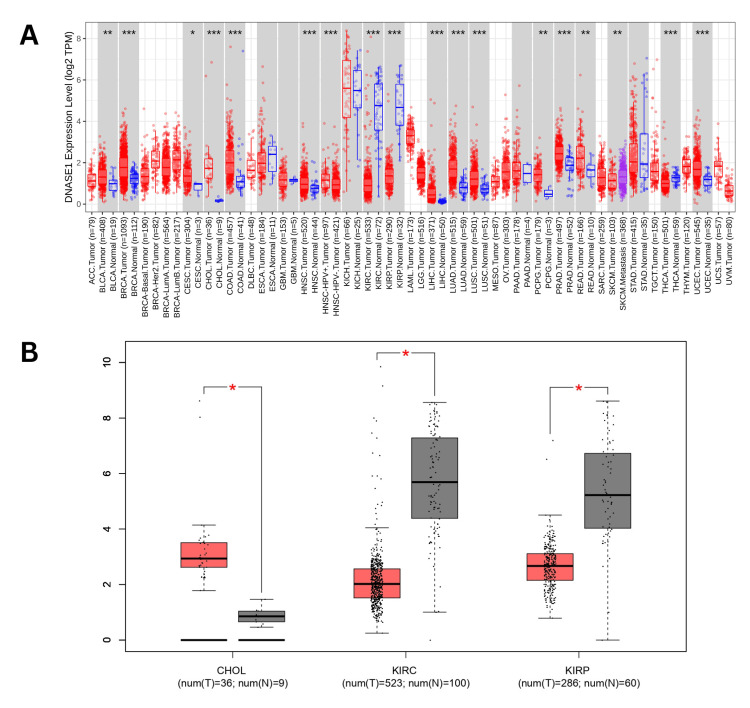
Pan-cancer analysis conducted to evaluate the expression of DNASE1 across various cancers. (A) Pan-cancer view using TIMER. (B) Pan-cancer view using GEPIA. *p < 0.05, **p < 0.01, and ***p < 0.001. DNASE1: deoxyribonuclease 1; TIMER: Tumor Immune Estimation Resource; p: p-value; GEPIA: Gene Expression Profiling Interactive Analysis; ACC: adrenocortical carcinoma; BLCA: bladder urothelial carcinoma; BRCA: breast invasive carcinoma; Her2: human epidermal growth factor receptor 2; LumA: luminal A; LumB: luminal B; CESC: cervical squamous cell carcinoma and endocervical adenocarcinoma; CHOL: cholangiocarcinoma; COAD: colon adenocarcinoma; DLBC: lymphoid neoplasm diffuse large B-cell lymphoma; ESCA: esophageal carcinoma; GBM: glioblastoma multiforme; HNSC: head and neck squamous cell carcinoma; HPV+: human papillomavirus positive; HPV–: human papillomavirus negative; KICH: kidney chromophobe; KIRC: kidney renal clear cell carcinoma; KIRP: kidney renal papillary cell carcinoma; LAML: acute myeloid leukemia; LGG: brain lower grade glioma; LIHC: liver hepatocellular carcinoma; LUAD: lung adenocarcinoma; LUSC: lung squamous cell carcinoma; MESO: mesothelioma; OV: ovarian serous cystadenocarcinoma; PAAD: pancreatic adenocarcinoma; PCPG: pheochromocytoma and paraganglioma; PRAD: prostate adenocarcinoma; READ: rectum adenocarcinoma; SARC: sarcoma; SKCM: skin cutaneous melanoma; STAD: stomach adenocarcinoma; TGCT: testicular germ cell tumors; THCA: thyroid carcinoma; THYM: thymoma; UCEC: uterine corpus endometrial carcinoma; UCS: uterine carcinosarcoma; UVM: uveal melanoma; num(T): the number of tumor samples; num(N): the number of normal samples.

The present study conducted a comprehensive analysis of deoxyribonuclease 1 (DNASE1) across various cancers using the TIMER. Our analysis revealed that the expression of DNASE1 was significantly upregulated in 13 cancers, including bladder urothelial carcinoma (BLCA), breast invasive carcinoma (BRCA), cervical squamous cell carcinoma (CESC), cholangiocarcinoma (CHOL), colon adenocarcinoma (COAD), head and neck squamous cell carcinoma (HNSC), liver hepatocellular carcinoma (LIHC), lung adenocarcinoma (LUAD), lung squamous cell carcinoma (LUSC), pheochromocytoma and paraganglioma (PCPG), prostate adenocarcinoma (PRAD), rectum adenocarcinoma (READ), and uterine corpus endometrial carcinoma (UCEC) compared to normal samples. However, DNASE1 expression was significantly downregulated in three cancers: KIRC, KIRP, and THCA (thyroid carcinoma) compared to normal samples. We also found that the expression of DNASE1 in skin cutaneous melanoma (SKCM) metastasis tissues was higher than that in SKCM tumor tissues, but there were no available data for comparing either SKCM tumor or metastasis tissues to normal tissues as of 2/11/2024 (Figure 1A). We further analyzed DNASE1 expression across 17 cancers obtained from TIMER using GEPIA and found that DNASE1 was overexpressed in CHOL (num(T)=36; num(N)=9), and kidney chromophore (KICH) (num(T)=66; num(N)=53), compared to normal samples, whereas DNASE1 was under-expressed in two cancers, including KIRC (num(T)=523; num(N)=100) and KIRP (num(T)=286; num(N)=60) (Figure 1B). Additionally, DNASE1 expression was not significantly different between BLCA, BRCA, CESC, COAD, HNSC, LIHC, LUAD, LUSC, PCPG, PRAD, READ, UCEC, THCA, and SKCM.

**Figure 2:**
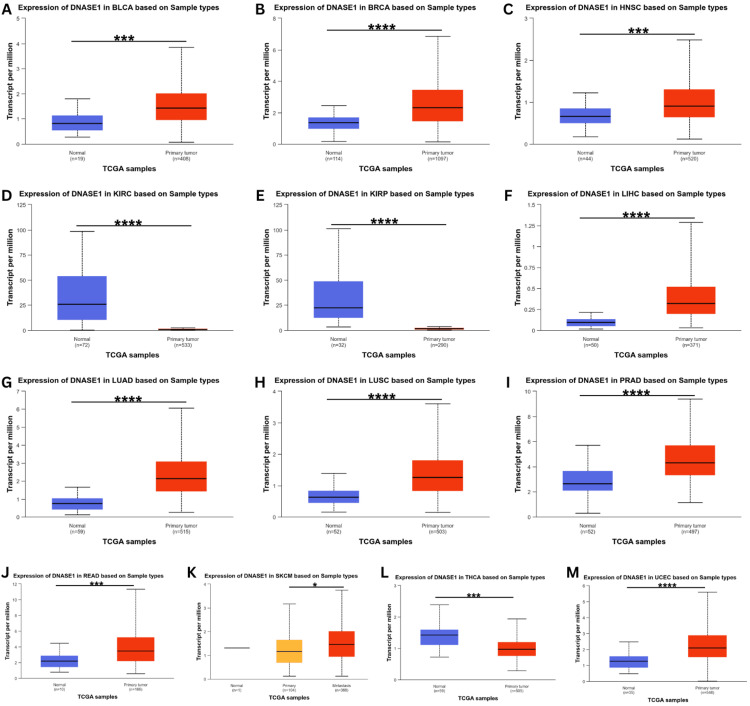
Illustration of expression profiles of DNASE1 across diverse cancers using the UALCAN database. (A) in BLCA, (B) in BRCA, (C) in HNSC, (D) in KIRC, (E) in KIRP, (F) in LIHC, (G) in LUAD, (H) in LUSC, (I) in PRAD, (J) in READ, (K) in SKCM, (L) in THCA, (M) in UCEC. *p < 0.05, ***p < 0.001, and ****p < 0.0001. DNASE1: deoxyribonuclease 1; UALCAN: University of Alabama at Birmingham Cancer Data Analysis Portal; p: p-value; BLCA: bladder urothelial carcinoma; BRCA: breast invasive carcinoma; HNSC: head and neck squamous cell carcinoma; KIRC: kidney renal clear cell carcinoma; KIRP: kidney renal papillary cell carcinoma; LIHC: liver hepatocellular carcinoma; LUAD: lung adenocarcinoma; LUSC: lung squamous cell carcinoma; PRAD: prostate adenocarcinoma; READ: rectum adenocarcinoma; SKCM: skin cutaneous melanoma; THCA: thyroid carcinoma; UCEC: uterine corpus endometrial carcinoma; TCGA: The Cancer Genome Atlas.

DNASE1 expression was assessed using the UALCAN database to validate its expression in the 17 cancer samples collected from the TIMER database. This analysis confirmed the upregulation of DNASE1 expression in BLCA, BRCA, HNSC, LIHC, LUAD, LUSC, PRAD, READ, and UCEC tissues (Figure 2). We also found that DNASE1 mRNA expression was significantly lower in KIRC, KIRP, and THCA tissues than in normal tissues and that DNASE1 expression was higher in SKCM metastasis than in SKCM tumors (Figure 2K). Furthermore, we included two cancers (KIRC and KIRP) that were agreed between the three databases (UALCAN, TIMER, GEPIA) in terms of DNASE1 expression for further analysis.

Clinical parameters analysis of DNASE1 expression

**Figure 3:**
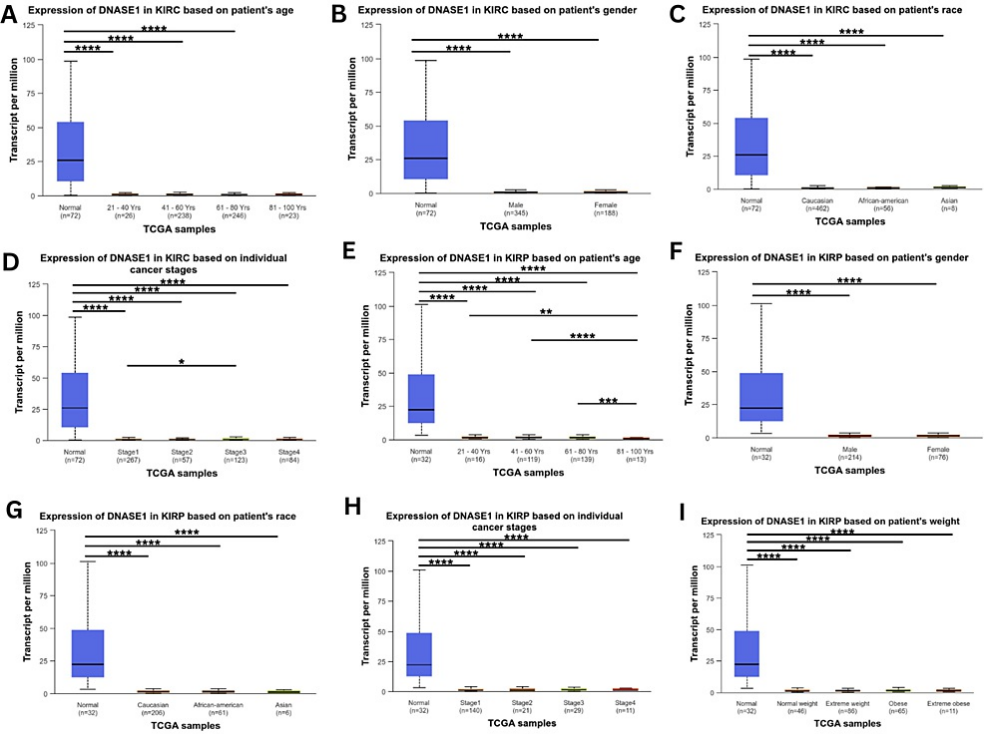
Exploration for identifying the significance of DNASE1 expression in cancer patients based on different clinical parameters (age, sex, race, cancer stage, and weight) using the UALCAN database. (A-D) DNASE1 expression in KIRC patients according to age, sex, race, and cancer stage. (E-I) The expression of DNASE1 in KIRP patients based on age, sex, race, cancer stage, and weight. *p < 0.05, **p < 0.01, ***p < 0.001, and ****p<0.0001. DNASE1: deoxyribonuclease 1; UALCAN: University of Alabama at Birmingham Cancer Data Analysis Portal; p: p-value; KIRC: kidney renal clear cell carcinoma; KIRP: kidney renal papillary cell carcinoma; TCGA: The Cancer Genome Atlas.

We analyzed the correlation between DNASE1 expression and clinicopathological parameters, such as age, sex, race, cancer stage, and weight, in two cancers that agreed between the three databases we previously mentioned (KIRC and KIRP), starting with age groups: young adults (21-40 years), middle-aged adults (41-60 years), older adults (61-80 years), and elders (81-100 years). Starting with KIRC, we found that DNASE1 expression in young adults (p = 1.66e-12), middle-aged adults (p = 1.48e-14), and older adults (p = 3.66e-15) was significantly lower than that in normal tissues, but gene expression was not significantly different among the four age groups. In addition, the expression was not significantly different between normal and elderly samples (Figure 3A). According to the sex (male and female) of KIRC patients, we noticed a higher expression of DNASE1 in both males (p = 1.66e-12) and females (p = 3.11e-15) than in normal samples; however, DNASE1 expression was not significantly different between males and females (Figure 3B). Based on the patients’ race (African American, Caucasian, and Asian) in KIRC, we found significantly lower expression of DNASE1 in Caucasians (p = 1.64e-12), African Americans (p = 1.63e-12), and Asians (p = 2.55e-15) than in normal samples, and DNASE1 expression was not statistically significant among the three race groups (Figure 3C). According to the individual cancer stages of patients with KIRC, DNASE1 was expressed lower in stage 1 (p = 1.64e-12), stage 2 (p = 2.14e-05), stage 3 (p = 2e-15), and stage 4 (p = 2e-15) than in normal tissues. Furthermore, DNASE1 was slightly under-expressed in stage 1 (p = 4.97e-02) compared to stage 3. Moreover, the expression of DNASE1 was not significantly different in stage 1 when compared to stages 2 and 4, and DNASE1 expression was not significantly different between stages 2, 3, and 4. There were no statistically significant differences between stages 3 and 4 (Figure 3D). However, when we analyzed DNASE1 expression in patients with KIRC according to patient weight (normal weight, extreme weight, obese, and extremely obese), there were no available datasets in the UALCAN database as of February 11, 2024.

For KIRP patients, we found low expression of DNASE1 in elders (p = 9.42e-08), middle-aged adults (p = 1.63e-07), young adults (p = 1.79e-07), and older adults (p = 2.03e-07) when compared to normal tissues, whereas elderly patients also had low expression of DNASE1 when compared to the other three age groups: young adults (p = 1.28e-03), middle-aged adults (p = 5.31e-07), and older adults (p = 8.84e-04). Gene expression in young patients was not statistically significant when compared to that in older and middle-aged patients, while there was no significant difference between middle-aged and older patients (Figure 3E). Based on the patient’s sex in KIRP, DNASE1 expression was relatively lower in male (p = 1.83e-07) and female patients (p = 1.58e-07) with KIRP than in normal samples, and DNASE1 expression was not significantly different between male and female patients (Figure 3F). According to patient’s race for KIRP patients, DNASE1 expression was significantly lower in Caucasians (p = 1.68e-07), African Americans (p = 2.24e-07), and Asians (p = 1.40e-07) than in normal tissues, while no significant difference in DNASE1 expression was observed among the three race groups (Figure 3G). Based on the individual cancer stages of KIRP patients, DNASE1 expression was lower in stage 1 (p = 1.93e-07), stage 2 (p = 1.75e-07), stage 3 (p = 1.61e-07), stage 4 (p = 1.98e-07) in KIRP patients than in normal tissues. However, we found no significant differences in the expression of DNASE1 among the four cancer stages (Figure 3H). Furthermore, based on the weight of KIRP patients, we found that the expression of DNASE1 was downregulated in normal weight (p = 2.20e-07), extreme weight (p = 1.40e-07), obese (p = 1.66e-07), and extremely obese (p = 2.38e-05) groups compared to normal samples; however, we found no significant differences among the four different body weight groups (Figure 3I).

DNASE1 is correlated with the abundance of immune cells

**Figure 4:**
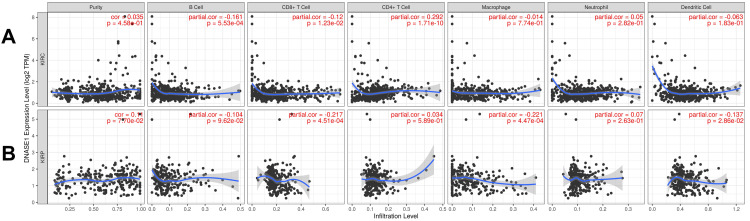
Correlation of DNASE1 expression with immune infiltration levels (B cells, CD8+ T cells, CD4+ T cells, macrophages, neutrophils, and dendritic cells) using the TIMER database. (A) KIRC. (B) KIRP. DNASE1: deoxyribonuclease 1; TIMER: Tumor Immune Estimation Resource; KIRC: kidney renal clear cell carcinoma; KIRP: kidney renal papillary cell carcinoma; B cells: B lymphocytes; CD8+ T cells: cytotoxic T lymphocytes; CD4+ T cells: T helper cells; cor: correlation; partial cor: partial correlation; p: p-value.

We investigated the correlation between DNASE1 expression and the abundance of immune cells, including B cells, CD8+ T cells, CD4+ T cells, macrophages, neutrophils, and dendritic cells, in addition to tumor purity, in the two cancers that we previously mentioned. In patients with KIRC, we found a negative correlation between DNASE1 expression and the abundance of B cells (Cor = -0.161, p = 5.53e-04) and CD8+ T cells (Cor = -0.12, p = 1.23e-02); however, there was a positive correlation between the expression of DNASE1 and infiltration of CD4+ T cells (Cor = 0.292, p = 1.71e-10) (Figure 4A). In KIRP, DNASE1 expression levels were negatively correlated with the infiltration of CD8+ T cells (Cor = -0.217, p = 4.51e-04), macrophages (Cor = -0.221, p = 4.47e-04), and dendritic cells (Cor = -0.137, p = 2.86e-02) (Figure 4B).

DNASE1 is correlated with the prognosis of patients with KIRC

**Figure 5:**
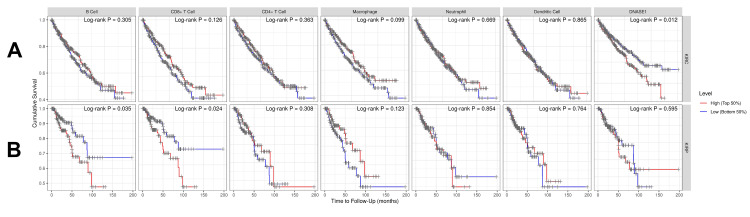
The correlation between immune cells (B cells, CD4+ T Cells, CD8+ T cells, macrophages, neutrophils, and dendritic cells), tumor purity, DNASE1 expression, and OS of patients using the TIMER database. (A) KIRC. (B) KIRP. DNASE1: deoxyribonuclease 1; TIMER: Tumor Immune Estimation Resource; B cells: B lymphocytes; CD8+ T cells: cytotoxic T lymphocytes; CD4+ T cells: T helper cells; OS: overall survival; KIRC: kidney renal clear cell carcinoma; KIRP: kidney renal papillary cell carcinoma; Log-rank P: p-value resulting from log-rank test.

We investigated the correlation between DNASE1 and the prognosis of patients with KIRC and KIRP using the TIMER database while adding different clinical parameters for the Cox proportional hazard model: tumor purity, age, sex, race, and stage. We found that high DNASE1 expression was associated with poor prognosis in patients with KIRC (p = 0.012; Figure 5A). However, for KIRP, DNASE1 expression was not significantly correlated with prognosis (Figure 5B). Moreover, the Cox proportional hazard model showed that age, stage 3, and stage 4 (all p = 0) were associated with poor prognosis in KIRC patients, while CD8+ T cells (p = 0.033) were correlated with favorable prognosis (Table 1). In KIRP, we discovered that stage 3 (p = 0.004), stage 4 (p < 0.001), B cells (p = 0.023), and CD8+ T cells (p = 0.040) were positively correlated with poor prognosis (Table 2).

**Table 1:** The Cox proportional hazard model of DNASE1, immune cells, and clinical parameters in KIRC for 415 patients with 123 dying. *p < 0.05, ***p < 0.001. DNASE1: deoxyribonuclease 1; Coef: coefficient; KIRC: kidney renal clear cell carcinoma; HR: hazard ratio; 95%CI_l: lower 95% confidential interval; 95%CI_u: upper 95% confidential interval; sig: significance; p: p-value.

Survival Factors	Coef	HR	95%CI_l	95%CI_u	p-value	sig
Purity	-0.153	0.858	0.357	2.063	0.733	-
Age	0.034	1.035	1.017	1.053	0	***
gender, male	-0.119	0.888	0.599	1.315	0.552	-
race, Black	0.203	1.225	0.143	10.523	0.853	-
race, White	0.143	1.154	0.147	9.032	0.892	-
Stage 2	0.418	1.519	0.755	3.056	0.241	-
Stage 3	0.886	2.426	1.499	3.925	0	***
Stage 4	1.856	6.401	4.001	10.24	0	***
B_cell	-1.598	0.202	0.004	11.441	0.438	-
CD8_Tcell	-2.051	0.129	0.02	0.845	0.033	*
CD4_Tcell	-0.204	0.816	0.026	25.639	0.908	-
Macrophage	-0.783	0.457	0.029	7.245	0.579	-
Neutrophil	0.806	2.24	0.01	497.982	0.77	-
Dendritic	1.35	3.857	0.391	38.047	0.248	-
DNASE1	0.1	1.105	0.899	1.359	0.343	-

**Table 2:** The Cox proportional hazard model of DNASE1, immune cells, and clinical parameters in KIRP for 214 patients with 34 dying. *p < 0.05, **p < 0.01, ***p < 0.001. DNASE1: deoxyribonuclease 1; Coef: coefficient; KIRP: kidney renal papillary cell carcinoma; HR: hazard ratio; 95%CI_l: lower 95% confidential interval; 95%CI_u: upper 95% confidential interval; sig: significance; p: p-value.

Survival Factors	Coef	HR	95%CI_l	95%CI_u	p-value	sig
Purity	0.804	2.233	0.286	17.454	0.444	-
Age	0.024	1.025	0.99	1.06	0.167	-
Gender, male	-0.104	0.901	0.37	2.193	0.819	-
Race, Black	-1.689	0.185	0.009	3.595	0.265	-
Race, White	-1.706	0.182	0.01	3.316	0.25	-
Stage 2	-0.22	0.803	0.091	7.084	0.843	-
Stage 3	1.312	3.714	1.53	9.016	0.004	**
Stage 4	2.781	16.13	5.528	47.063	0	***
B_cell	7.585	1968.028	2.78	1393129	0.023	*
CD8_Tcell	9.15	9418.654	1.503	59005503	0.04	*
CD4_Tcell	0.457	1.579	0.001	2518.155	0.903	-
Macrophage	-2.452	0.086	0	101.798	0.497	-
Neutrophil	2.16	8.667	0	35195785	0.781	-
Dendritic	-2.054	0.128	0.001	18.594	0.419	-
DNASE1	0.163	1.177	0.703	1.973	0.535	-

DNASE1 is a prognostic biomarker in KIRC

**Figure 6:**
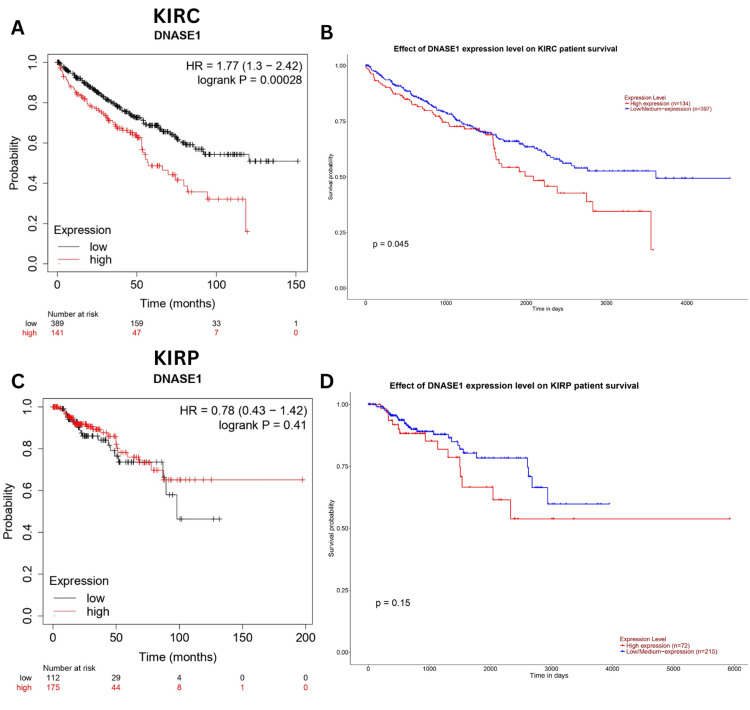
The correlation between the DNASE1 expression level and the survival outcome of patients using UALCAN and Kaplan-Meier Plotter databases. (A) The Kaplan-Meier Plotter database was used to investigate the relationship between DNASE1 expression and the overall survival of patients with KIRC. (B) The UALCAN database was used to validate the results of the UALCAN database for patients with KIRC in terms of overall survival. (C) The relationship between DNASE1 expression in patients with KIRP and overall survival using the Kaplan-Meier Plotter database. (D) Utilizing the UALCAN database, we demonstrated a correlation between the overall survival of KIRP patients and the expression of DNASE1. DNASE1: deoxyribonuclease 1; UALCAN: University of Alabama at Birmingham Cancer data analysis Portal; KIRC: kidney renal clear cell carcinoma; KIRP: kidney renal papillary cell carcinoma; HR: hazard ratio; logrank P: p-value resulting from log-rank test; p: p-value.

We explored the correlation between DNASE1 expression and patient outcomes in KIRC and KIRP using the Kaplan-Meier and UALCAN databases. We found that high expression of DNASE1 was associated with poor prognosis in KIRC patients (Kaplan-Meier, HR = 1.77, p = 0.00028, Figure 6A; UALCAN, p = 0.045, Figure 6B); however, there was no significant correlation between DNASE1 expression and the prognosis of KIRP patients in both databases (Figure 6C, D).

Genetic alteration analysis of DNASE1

**Figure 7:**
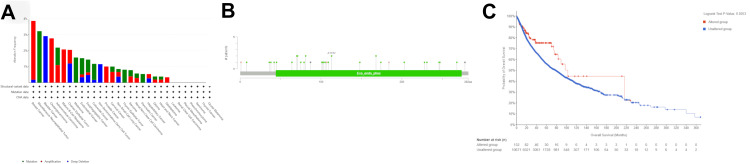
Genetic alteration analysis of DNASE1 using the cBioPortal database. (A) Alteration frequency of DNASE1 in various cancers. (B) Mutation sites in DNASE1 in various cancers using TCGA PanCancer Atlas Studies. (C) Correlation between the survival rate of patients with different types of cancer and genetic alterations in DNASE1. DNASE1: deoxyribonuclease 1; TCGA: The Cancer Genome Atlas; A113V (A: amino acid alanine; 113: amino acid position in the protein sequence; V: amino acid valine): represents the change in amino acid alanine to valine at position 113 of the protein sequence; Exo_endo_phos: endonuclease/exonuclease/phosphatase family.

We analyzed genetic variation in DNASE1 across various cancers using the cBioPortal platform. Utilizing TCGA datasets, we determined that DNASE1 was mutated in 1.2% of the queried samples (10,967 samples from 32 studies). Most DNASE1 mutations are amplification mutations, followed by deep deletions. We found that the majority of DNASE1 mutations occurred in breast cancer (amplification frequency = 3.68% (40 cases), deep deletion frequency = 0.18% (two cases); Figure 7A). DNASE1 expression was not mutated in most of the samples. We also found that the missense mutation for DNASE1 was dominant and position A113V had the most mutations across 10,953 samples for UCEC (mutation samples = 9,686) and stomach adenocarcinoma (STAD, mutation samples = 1,144) (Figure 7B). As shown in Figure 7C, patients with mutated DNASE1 had a better prognosis than those with non-mutated DNASE1 (p = 0.0253).

Integration of public datasets for cross-validation

**Figure 8:**
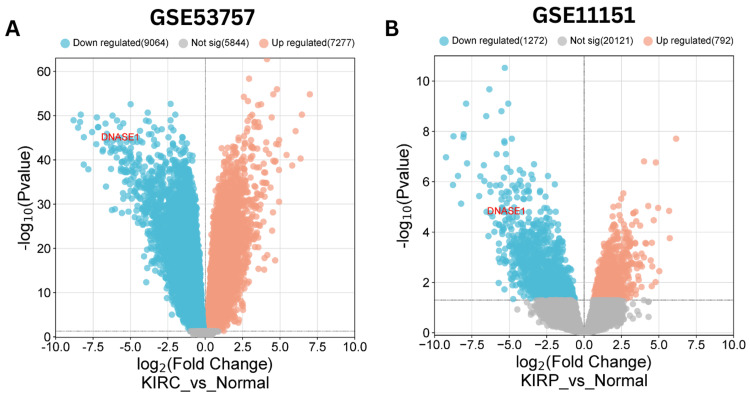
Volcano plots illustrate differential gene expression, including DNASE1, in KIRC and KIRP using the bioinformatics.com.cn platform. Upregulated genes are depicted as red dots, while downregulated genes are presented as blue dots (|Log2FC| >1, adj p-value < 0.05). (A) KIRC. (B) KIRP. DNASE1: deoxyribonuclease 1; KIRC: kidney renal clear cell carcinoma; KIRP: kidney renal papillary cell carcinoma; Log2FC: log2FoldChange; adj p-value: adjusted p-value; GSE53757: Gene Expression Omnibus Series with a unique identifier for kidney renal clear cell carcinoma datasets; GSE11151: Gene Expression Omnibus Series with a unique identifier for kidney renal papillary cell carcinoma datasets.

We validated the expression of DNASE1 in KIRC and KIRP using public datasets obtained from the Gene Expression Omnibus (GEO). We employed the GEO2R tool to indicate the differential expression of DNASE1 (|Log2FC| > 1, adjusted p-value < 0.05) and utilized the bioinformatics.com.cn platform to visualize the volcano plot for differentially expressed genes. The public datasets used were GSE53757 for KIRC and GSE11151 for KIRP. For the GSE53757 dataset, the normal group comprised 72 samples of healthy kidney tissue, whereas the KIRC group contained 72 samples of clear cell RCC across four stages. For the GSE11151 dataset, the KIRP group included 19 samples of papillary renal cell cancer, whereas the normal group included three adult normal kidney samples and two fetal normal kidney samples. Our analysis revealed 16,341 differentially expressed genes (DEGs) (7,277 upregulated and 9,064 downregulated genes) in KIRC (Figure 8A) and 2,064 DEGs (792 upregulated and 1,272 downregulated genes) in KIRP (Figure 8B). In summary, DNASE1 expression was downregulated in two cancers, namely, KIRC and KIRP. Whereas in 12 cancers (BRCA, BLCA, HNSC, LIHC, LUAD, LUSC, PRAD, READ, SKCM, UCEC, CHOL, and THCA), further investigation is required.

## Discussion

We utilized the TIMER, GEPIA, and UALCAN datasets to explore the expression pattern of DNASE1 across various malignancies, aiming to determine significant differences in DNASE1 expression between normal and tumor samples. According to the TIMER database, DNASE1 is significantly downregulated in KIRC and KIRP compared to normal samples. The downregulation of DNASE1 in kidney carcinoma suggests a role in disease development [15]. This downregulation may result in the inability to adequately digest extracellular DNA, leading to the accumulation of genetic material that can promote tumor development and metastasis. The imbalance in DNASE1 levels may affect the immune response in kidney cancer patients, as DNASE1 is involved in the clearance of apoptotic cells and immunological modulation by degrading extracellular DNA. Therefore, DNASE1 dysregulation in kidney cancer may impede the body's ability to properly clear apoptotic cells and control immunological responses, resulting in a compromised immune system and potentially accelerating tumor development and spread [16].

In GEPIA, DNASE1 was under-expressed in KIRC and KIRP compared to the expression levels in normal samples. In UALCAN, the mRNA expression levels of DNASE1 were also lower in KIRC and KIRP than in normal tissues, demonstrating consistency across the three databases for KIRC and KIRP.

We also used UALCAN to investigate the association between DNASE1 expression and clinicopathological parameters, including age, sex, race, individual cancer stage, and weight, focusing on two cancers: KIRC and KIRP. We found that the expression level of DNASE1 in normal samples was significantly upregulated in terms of age, sex, race, and cancer stage compared to that in KIRC and KIRP. We investigated the expression of DNASE1 across different age groups. One study found that DNASE1 expression levels tended to decrease with age [17]. Another study observed that DNASE1 expression was the highest during early development and gradually decreased throughout adulthood [18]. According to one study, Africans have higher levels of DNASE1 expression than Europeans. This heterogeneity may have substantial consequences in understanding immune response and disease susceptibility [19]. However, it should be highlighted that this is only one study, and further research is required to properly understand the degree of changes in gene expression among races, as well as their ramifications.

We identified an association between DNASE1 expression and the abundance of immune cells (macrophages, CD8+ T cells, CD4+ T cells, neutrophils, and B cells). Studies have found a link between macrophages and DNASE1 in tumor cells. These studies discovered that macrophages can produce DNASE1, which degrades extracellular DNA in the tumor microenvironment [20]. The degradation of extracellular DNA by DNASE1 produced by macrophages is expected to have significant implications for tumor growth and metastasis [21]. Next, we established the relationship between DNASE1 expression and the survival outcomes of cancer patients using TIMER, UALCAN, and the Kaplan-Meier plotter. We found that high expression of DNASE1 in KIRC was associated with poor prognosis in the three databases, whereas for KIRP, no correlation has been observed in the prognosis of patients in the three databases, establishing that DNASE1 can be a potential prognostic biomarker for KIRC patients. Additionally, we identified an association between DNASE1 genetic alterations and poor prognosis in 32 cancers using TCGA datasets obtained from the cBioPortal database. Finally, public datasets were used to validate DNASE1 expression in various types of cancer. We found a significant downregulation of DNASE1 expression in KIRC and KIRP compared to that in normal tissues, establishing the potential use of DNASE1 as a diagnostic biomarker in these cancers.

DNASE1 is associated with cancer development. Reports have revealed that DNASE1 treatment results in decreased metastasis and cancer development in the lungs and liver [22]. Furthermore, DNASE1 is involved in controlling cfDNA production and removal and plays a major role in tumorigenesis [23]. Cancer cells treated with recombinant DNases, including DNASE1, initiate programmed cell death [24]. A previous study observed a link between DNASE1 and thrombophilia by degrading neutrophil extracellular traps (NETs) in colorectal cancer, which could potentially lead to cancer-related coagulation problems [25].

There has been ongoing research on the correlation between DNASE1 and cancer; however, its mechanism of action remains unclear. Studying the expression of DNASE1 in cancer is crucial for understanding cancer pathways, and disturbances in DNASE1 can contribute to autoimmune diseases, potentially increasing the risk of developing cancer. Exploring DNASE1 expression in various cancers is important for the potential identification of its use in the early detection of cancer and the evaluation of clinical outcomes of patients.

Our study is the first pan-cancer analysis of DNASE1. We provide a more in-depth analysis of DNASE1 expression across various cancers to understand its role and how it affects cancer progression, which provides insight for future investigations, particularly in early cancer detection and the identification of genetic alterations in DNASE1 in patients. Our findings establish a starting point for exploring the correlation between DNASE1 expression and immune cell abundance in various cancers.

Our study has some limitations. This study relied on computational data, and further experimental wet lab analysis is needed to validate our results and to identify how DNASE1 expression affects the survival outcome of patients with KIRC as well as its correlation with immune infiltration.

## Conclusions

Our analysis revealed that DNASE1 could potentially be used as a diagnostic and prognostic biomarker in KIRC. In addition, we identified the potential use of this gene as a diagnostic biomarker for KIRP. This study was significant in establishing an association between DNASE1 expression and immune cell abundance in KIRC and KIRP. We conducted the first pan-cancer analysis on this gene, and our results will shed light on further studies and enable in-depth analysis of DNASE1, accompanied by wet lab research.
